# Case Report: When peripheral neuropathy meets hoarseness and cough: a diagnostic challenge and insights from a case of late-onset ATTRv

**DOI:** 10.3389/fmed.2026.1896915

**Published:** 2026-07-17

**Authors:** Lei Chen, Ling Zhu, Ye Deng, Ying Yuan, Wei Zhang, Yuting Fan, Long Luo

**Affiliations:** Department of Neurology, The Central Hospital of Xiangtan (The Affiliated Hospital of Hunan University), Xiangtan, Hunan, China

**Keywords:** case report, cough, hoarseness, p.Ala117Ser mutation, transthyretin amyloidosis

## Abstract

Hereditary transthyretin amyloidosis (ATTRv) is a progressive, life-threatening disease caused by mutations in the *TTR* gene. It is characterized by marked clinical heterogeneity, and diagnosis is often delayed. Here, we report the tortuous diagnostic process in a patient with late-onset ATTRv carrying a c.349G > T (p.Ala117Ser) mutation in the *TTR* gene. A 59-year-old man initially presented with distal numbness and weakness in both lower limbs, followed by hoarseness and cough. Over two years, he consulted multiple specialists for these predominant symptoms, but no definitive diagnosis was made. Electrocardiography revealed low limb lead voltages. Echocardiography showed increased echogenicity in the left ventricular posterolateral wall with wall thickness at the upper normal limit. With a strong clinical suspicion of transthyretin amyloidosis, 99mTc-pyrophosphate (PYP) scintigraphy demonstrated cardiac amyloid deposition, and the diagnosis was ultimately confirmed by genetic testing, which identified a heterozygous Ala117Ser mutation in the *TTR* gene. This case illustrates that in middle-aged and older patients with progressive peripheral neuropathy of unclear cause, atypical symptoms such as hoarseness and cough may serve as important clues to ATTRv. Prompt integration of cardiac imaging and genetic screening can help avoid diagnostic delay.

## Introduction

Hereditary transthyretin amyloidosis (ATTRv) is a progressive, fatal disease caused by mutations in the *TTR* gene, predominantly affecting the peripheral nerves and the heart (1, 2). Over 140 mutations have been reported worldwide, with clinical manifestations closely linked to the specific mutation type (3, 4). The p.Ala117Ser mutation is a distinctive subtype in the Chinese population, showing significant geographic clustering (southeastern coastal regions of mainland China and Taiwan) and typically presenting as a late-onset disorder with male predominance and frequent cardiac involvement (5, 6).

Nevertheless, the p.Ala117Ser mutation often has an insidious onset. Early manifestations may be limited to non-specific sensorimotor neuropathy without prominent autonomic dysfunction, rendering it easily misdiagnosed as chronic inflammatory demyelinating polyneuropathy (CIDP) or cryptogenic axonal polyneuropathy (CIAP) (3, 7). Importantly, respiratory symptoms such as dry cough have recently been recognized as an early, atypical presentation of the p.Ala117Ser mutation (7, 8); isolated hoarseness due to this mutation has not been separately reported. A lack of clinical awareness of these atypical features leads patients to consult otolaryngologists or pulmonologists, diverting attention from the primary diagnosis and prolonging the diagnostic process. This report describes a patient with late-onset ATTRv due to the p.Ala117Ser mutation, highlighting the diagnostic challenges, atypical clinical features, and the critical importance of early targeted investigations.

### Case presentation

A 59-year-old Han Chinese man presented with a two-year history of insidious-onset numbness in both feet, accompanied by a sensation of weakness. Symptoms waxed and waned, and he remained able to work. He had been evaluated at a local hospital for “peripheral neuropathy,” but treatment with B vitamins was ineffective. One year prior to admission, he developed new-onset hoarseness with a foreign-body sensation in the throat. He visited an otolaryngologist on multiple occasions; laryngoscopy revealed no organic lesion. Concurrently, he experienced recurrent nocturnal dry cough and consulted a pulmonologist several times; chest CT showed no significant abnormality; and antitussive medications were ineffective. Over the same period, the sensory level of numbness progressed to below the knees, accompanied by difficulty squatting and standing, and a “cotton-wool” sensation when walking. He also developed diarrhea, abdominal distension, constipation, increased urinary frequency, erectile dysfunction, sleep disturbance, and anxiety. He reported no obvious chest tightness,shortness of breath, nausea, or vomiting. His food intake was mildly reduced, and he experienced unintentional weight loss exceeding 5 kg over the preceding year. Gastrointestinal symptoms improved after treatment with mosapride.

He had no significant chronic medical history. He had a 30 -year smoking history but had quit more than one year previously. He denied alcohol use or toxin exposure. His parents were deceased (cause unknown). One of his three older brothers had “unexplained weight loss” but refused evaluation. A sister and other family members in the third and fourth generations were reportedly healthy.

**Figure 1:**
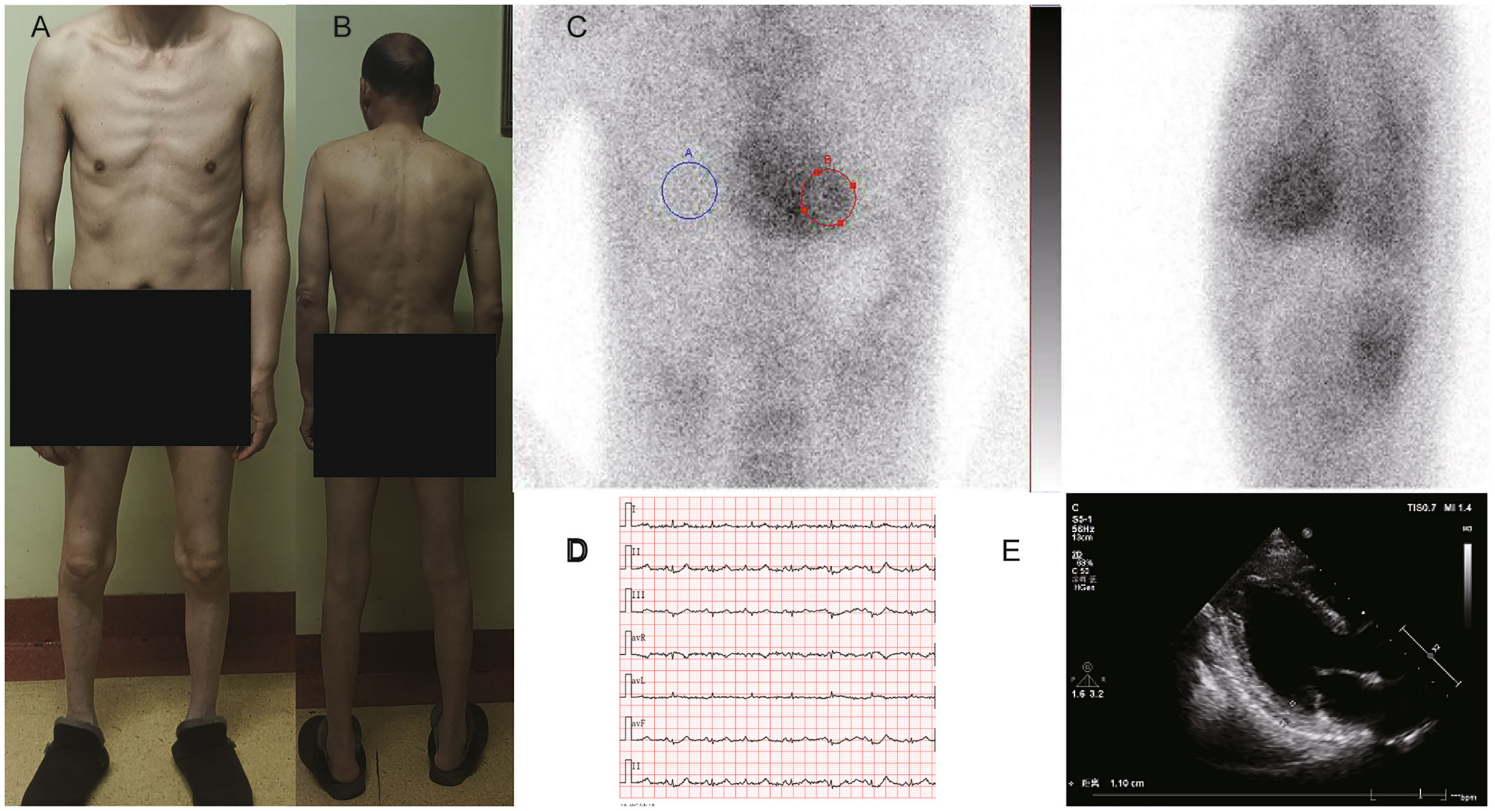
Clinical and paraclinical features of the patient. **(A,B)** Anterior and posterior views showing lean body habitus and muscle atrophy in the limbs. **(C)** 99mTc-PYP cardiac scintigraphy (anterior and left lateral views) demonstrating myocardial uptake at 1 h that is clearly greater than rib uptake; the heart-to-contralateral (H/CL) ratio was 1.78. **(D)** Electrocardiography showing low limb lead voltages. **(E)** Echocardiography showing increased echogenicity in the left ventricular posterolateral wall with wall thickness at the upper normal limit.

Neurological examination revealed a lean body habitus with muscle atrophy in the limbs (Figures 1A,B). He was alert but had hoarseness. Pupils were equal, round, and reactive to light. Neurological examination showed a glove-and-stocking pattern of hypoesthesia to pinprick; reduced vibration sense at the radial styloid processes and ankles; muscle strength grade 4/5 in all four limbs; absent deep tendon reflexes; and negative Babinski signs bilaterally. His gait was wide-based. Nerve conduction studies showed a bilateral, symmetric, predominantly axonal, length-dependent sensorimotor peripheral neuropathy, more severe in the lower than upper limbs (detailed in Supplementary material). Routine laboratory tests, including complete blood count, renal and liver function, glucose, lipids, vitamin B12, folate, thyroid function, and infectious disease screening, were unremarkable (detailed in Supplementary Table 1). Electrocardiography demonstrated sinus rhythm with low limb lead voltages (Figure 1D). Echocardiography showed increased echogenicity in the left ventricular posterolateral wall, with a wall thickness of 11 mm (reference range 6–11 mm) and grade I diastolic dysfunction. Myocardial strain analysis suggested reduced basal strain (Figure 1E). Cardiac magnetic resonance imaging was suggested but declined by the patient. Chest CT and abdominal ultrasound were normal. Autonomic function assessment revealed orthostatic blood pressure changes (supine 123/82 mmHg, standing 99/73 mmHg), and a COMPASS-31 score of 33, indicating autonomic dysfunction.

Comprehensive neuropsychological and functional scale assessments were performed. The Mini-Mental State Examination (MMSE) score was 28/30 (normal cognition). The Hamilton Anxiety Rating Scale score was 20/56 (indicating anxiety); the Hamilton Depression Rating Scale score was 21/76 (indicating depression). The overall disability sum score for RODS was 45/48 (good activities of daily living). The Inflammatory Neuropathy Cause and Treatment (INCAT) disability scale score was 3/10 (mild disability). The INCAT sensory sum score (ISS) was 2/20 (mild sensory deficit). The Timed Up and Go (TUG) test averaged 28.9 s (moderately impaired mobility with fall risk). Polyneuropathy Disability (PND) score was stage II (ambulatory without assistive device). The Norfolk Quality of Life–Diabetic Neuropathy (QOL-DN) total score was 29/136 (mildly affected quality of life).

Given the subacute progressive symmetric peripheral neuropathy in a 59-year-old man, starting in the lower limbs and involving both motor and sensory fibers with axonal predominance on electrodiagnostic studies, several etiologies were considered. Chronic inflammatory demyelinating polyneuropathy was considered less likely given the absence of typical demyelinating features. Paraneoplastic neuropathy was possible given the weight loss, but no tumor was identified. Cryptogenic axonal polyneuropathy remained a diagnosis of exclusion. Diabetic neuropathy was unlikely given no history of diabetes and the severity of neuropathy disproportionate to typical diabetic polyneuropathy. AL amyloidosis was excluded by negative serum and urine immunofixation electrophoresis and normal serum free light chain ratio (*κ*/*λ* = 1.25). Axonal Charcot–Marie–Tooth disease (CMT2) can present with a similar phenotype but usually manifests in adolescence or early adulthood; late-onset (>50 years) is rare. Given the high suspicion for transthyretin amyloidosis (ATTR)—despite the absence of cardiac complaints—the presence of low limb lead voltages and structural cardiac abnormalities on echocardiography prompted further evaluation. Lumbar puncture, tissue biopsy, and 99mTc-PYP cardiac scintigraphy were recommended for diagnostic confirmation, but the patient declined invasive procedures. 99mTc-PYP scintigraphy showed intense myocardial tracer uptake (Figure 1\C) with a heart-to-contralateral (H/CL) ratio of 1.78 and a visual score of grade 3, meeting the diagnostic criteria for ATTR cardiac amyloidosis. Whole-exome sequencing ultimately confirmed a heterozygous c.349G > T (p.Ala117Ser) mutation in the *TTR* gene. The diagnosis of ATTRv, mixed phenotype (polyneuropathy with cardiomyopathy), with possible involvement of bulbar muscles or vocal cords, was established.

After diagnosis, treatment options were discussed with the patient and his family. Current guideline-recommended first-line therapies include tafamidis and acoramidis (for cardiomyopathy) and the gene-silencing agents patisiran, vutrisiran, and inotersen (for polyneuropathy). However, these medications are expensive and unaffordable for the patient. After discussing the potential benefits and risks of alternative therapies and obtaining written informed consent, the following regimen was initiated: diflunisal 250 mg twice daily to stabilize the TTR tetramer, combined with doxycycline 100 mg twice daily and tauroursodeoxycholic acid 250 mg three times daily to promote clearance of amyloid fibrils (9). Trazodone 50 mg nightly was prescribed for sleep and mood disturbances. The patient tolerated the regimen well. At three-month follow-up, he reported no significant change in lower limb numbness, hoarseness, or cough, but sleep and mood had improved. Long-term outcomes require continued observation.

## Discussion

Amyloidosis encompasses a group of diseases resulting from the extracellular deposition of misfolded proteins, and more than 30 pathogenic amyloid proteins have been identified to date (1). In a consecutive series of over 7,000 patients at the UK National Amyloidosis Centre, the proportions of AL, AA, ATTRv, wild-type ATTR, and localized amyloidosis were 60, 10, 10, 8, and 10%, respectively (10). ATTRv amyloidosis is primarily caused by mutations in the *TTR* gene, which reduce the stability of the transthyretin tetramer, leading to its dissociation and misfolding. This process ultimately results in the formation of insoluble amyloid fibrils that deposit in multiple organs (11), most commonly affecting the peripheral nerves and the heart (2). With the advent of disease-modifying therapies, early diagnosis of ATTRv has become critically important (12, 13).

The p.Ala117Ser mutation is a distinctive subtype in the Chinese population with notable geographic clustering. Multiple studies have confirmed that this mutation predominantly clusters in the southeastern coastal provinces of mainland China (including Fujian, Guangdong, and Hunan) as well as Taiwan (5, 6, 14–16). An analysis of 57 Taiwanese ATTRv kindreds by Chao et al. (17) demonstrated that the p.Ala117Ser mutation accounted for 91.2% of all families. The mean age of onset was 58.2 ± 7.2 years, males accounted for 69.6% of cases, and approximately half of the patients initially presented with carpal tunnel syndrome. A cohort of 21 patients with the p.Ala117Ser mutation in southern China (male-to-female ratio 18:3, mean onset age 56.5 ± 7.2 years) reported by Zhu et al. (5) showed that 71.4% presented with limb numbness as the initial symptom, and the incidences of autonomic dysfunction (85.7%) and cardiac involvement (80.9%) were extremely high. The study by Wang et al. (15) further confirmed that Ala97Ser is the most frequent mutation (40%) among patients with ATTRv cardiomyopathy in southern China, with the majority exhibiting a mixed phenotype (82.8%) and a median post-diagnosis survival of 47.6 months. The present patient——a 59-year-old man presenting with limb weakness, numbness, gastrointestinal symptoms, orthostatic hypotension, and a positive cardiac PYP scintigraphy——is highly consistent with these clinical profiles of the p.Ala117Ser mutation in the Chinese population. However, the literature on respiratory symptoms associated with the p.Ala97Ser mutation remains limited. Although a few reports have described dyspnea (14), hoarseness or chronic cough has rarely been documented—which may reflect a genuinely low incidence, or alternatively, may be due to the fact that previous studies did not systematically observe such symptoms.

Respiratory symptoms are not uncommon in amyloidosis. The literature indicates that approximately 50% of patients with amyloidosis may develop respiratory tract involvement, which can be classified by deposition site into tracheobronchial, nodular parenchymal, and diffuse alveolar septal patterns (18, 19). The larynx is the most commonly affected site in localized amyloidosis of the head and neck region. Amyloid deposition can be observed in approximately 0.5–1% of benign laryngeal diseases, typically presenting clinically as hoarseness or stridor (20, 21). However, cases of ATTRv with prominent hoarseness as a key manifestation are extremely rare in the literature. Yuan et al. (7) previously reported a patient carrying the same p.Ala117Ser mutation whose rare presentation was chronic paroxysmal dry cough. The present case, featuring both dry cough and hoarseness, further expands the clinical spectrum of respiratory symptoms associated with the p.Ala117Ser mutation.

The precise mechanisms underlying hoarseness and cough in this context remain unclear, but several possibilities can be postulated: (1) Focal laryngeal amyloid deposition, where sub-mucosal deposition of amyloid in the supraglottic region causes vocal cord dysfunction and hoarseness (21); (2) Involvement of the vagus nerve or recurrent laryngeal nerve, as amyloid can infiltrate cranial nerves and it has been demonstrated that the Ile127Val mutation can damage the tenth cranial nerve (vagus nerve), manifesting as vocal cord paralysis and dysphagia (22); and (3) Tracheobronchial amyloid deposition, which can induce airway hyperresponsiveness and chronic cough (7, 19). The absence of any overt lesion on laryngoscopy in this patient lends support to a neurogenic mechanism, specifically the infiltration of the recurrent laryngeal nerve or the laryngeal branches of the vagus nerve by amyloid.

The diagnosis of ATTRv is often delayed. According to published data, the average delay from symptom onset to diagnosis is approximately 4 years for patients presenting initially with peripheral neuropathy and can be as long as 8 years for those presenting with cardiomyopathy (23). The diagnostic odyssey of the present case spanned approximately two years from initial symptom onset to final diagnosis, involving consultations across neurology, otolaryngology, pulmonology, and gastroenterology, without a definitive diagnosis being reached. The reasons for this diagnostic difficulty are multifactorial: the rarity of ATTRv, the non-specific nature of its early symptoms, and a lack of awareness among clinicians. When patients present with atypical complaints such as hoarseness and cough, the diagnostic process is easily diverted to other specialties (24); indeed, many patients consult five or more physicians before receiving a correct diagnosis (25).

This diagnostic dilemma highlights the value of precision medicine—achieving early and accurate diagnosis through the integration of multidimensional evidence from atypical clues. TTR stabilizers such as tafamidis are most effective when initiated early in the course of the disease (12, 13), as they can delay the progression of neuropathy, preserve cardiac function, and prolong survival. Furthermore, ATTRv follows an autosomal dominant inheritance pattern. Once a proband is diagnosed, their immediate family members can undergo genetic testing and counseling, enabling presymptomatic diagnosis and early intervention (26).

ATTRv imposes a significant psychological burden on patients and their families (27). Spouses of patients with ATTRv cardiomyopathy typically assume the role of caregivers and consequently experience considerable anxiety. For patients with ATTRv polyneuropathy, stress arises not only from the physical consequences of the disease itself but also from witnessing the illness in parents and other relatives, as well as concerns about potentially transmitting the pathogenic mutation to their children [28]. Therefore, we urge clinicians to pay adequate attention to the mental health of both patients and their families during the diagnostic and therapeutic process and to provide appropriate psychological support and genetic counseling when needed.

## Limitations

This study has several limitations. First, the patient declined lumbar puncture and tissue biopsy, so histologic confirmation of amyloid deposition was not obtained. Regarding the exact etiology of hoarseness and cough, although repeated laryngoscopy showed no organic lesion, the patient refused bronchoscopy and laryngeal electromyography; therefore, the precise mechanism (focal laryngeal deposition, vagus/recurrent laryngeal nerve involvement, or tracheobronchial deposition) remains speculative, based only on clinical features. Second, for financial reasons, the patient did not receive TTR stabilizer or gene-silencing therapy; treatment response will require longer follow-up. Third, the patient refused TTR genetic screening for his immediate family members, precluding assessment of disease risk in other potential mutation carriers and preemptive early intervention. Finally, as a single case report, the generalizability of our observations is limited.

### Patient perspective

For two years, I repeatedly visited neurologists for weakness and numbness in my lower limbs, otolaryngologists for hoarseness, pulmonologists for cough, and gastroenterologists for abdominal distension, diarrhea, and constipation. I tried many Western and Chinese herbal medicines, but my symptoms continued to worsen. Although doctors had analyzed my condition, when one suggested cardiac tests, I was confused—why examine my heart for a leg problem? Only when the results came back did I understand the connection. Subsequent genetic testing finally provided a diagnosis. Looking back on this tortuous journey, I sincerely hope my experience can help other patients with similar symptoms avoid misdiagnosis, inappropriate treatment, and delays in receiving a correct diagnosis.

## Conclusion

This report describes a patient with late-onset, mixed phenotype ATTRv due to the p.Ala117Ser mutation in the *TTR* gene. The presentation was insidious and included unusual features such as hoarseness and cough, leading to referrals across multiple specialties. For middle-aged and older patients with progressive peripheral neuropathy of unexplained cause, clinicians should maintain a high index of suspicion for ATTRv and recognize that hoarseness and cough may be part of the clinical spectrum. Even in the absence of cardiac complaints, electrocardiography and echocardiography should be actively performed. 99mTc-PYP scintigraphy and *TTR* genetic testing can facilitate early recognition and diagnosis, offering patients the opportunity for timely intervention.

## Data Availability

The original contributions presented in the study are included in the article/Supplementary material, further inquiries can be directed to the corresponding author/s.
