# Supplementary material for: Case Report: When peripheral neuropathy meets hoarseness and cough: a diagnostic challenge and insights from a case of late-onset ATTRv
Source: Front Med (Lausanne). 2026 Jul 17;13:1896915. doi: 10.3389/fmed.2026.1896915 (PMC13425136; doi:10.3389/fmed.2026.1896915)
Supplement: Supplementary file 1 [file Table_1.docx]

Supplementary Table 1 laboratory findings

| **Laboratory Test** | **Result** | **Reference Range** |
| --- | --- | --- |
| **Routine blood tests** |  |  |
| White blood cell count (WBC) | 8.32 × 10⁹/L | 3.97–9.15 × 10⁹/L |
| Neutrophil count (NEU) | 6.79 × 10⁹/L | 2.0–7.0 × 10⁹/L |
| Lymphocyte count (LYM) | 1.0 × 10⁹/L | 0.8–4.0 × 10⁹/L |
| Lymphocyte percentage (LYM%) | **12.3% ↓** | 20.0–40.0% |
| Hemoglobin (HGB) | 135.0 g/L | 120–160 g/L |
| Platelet count (PLT) | 195.0 × 10⁹/L | 85.0–303.0 × 10⁹/L |
| **Biochemical tests** |  |  |
| Alanine aminotransferase (ALT) | 16.9 IU/L | 0.0–40.0 IU/L |
| Aspartate aminotransferase (AST) | 17.2 IU/L | 0.0–45.0 IU/L |
| Creatinine (CREA) | 72.0 μmol/L | 53.0–115.0 μmol/L |
| Urea | 6.20 mmol/L | 2.90–8.20 mmol/L |
| Uric acid (UA) | 283.0 μmol/L | 89.0–430.0 μmol/L |
| Glucose (GLU) | 5.50 mmol/L | 3.90–6.10 mmol/L |
| Triglycerides (TG) | 0.81 mmol/L | < 1.70 mmol/L |
| Total cholesterol (CHOL) | 5.08 mmol/L | 2.90–5.20 mmol/L |
| High-density lipoprotein (HDL) | 1.49 mmol/L | > 1.04 mmol/L |
| Low-density lipoprotein (LDL) | 2.48 mmol/L | < 3.4 mmol/L |
| Homocysteine (HCY) | 6.70 μmol/L | 4.0–15.4 μmol/L |
| **Cardiac markers** |  |  |
| High-sensitivity troponin T (hs-TnT) | **0.027 ng/mL ↑** | 0.003–0.014 ng/mL |
| N-terminal pro-B-type natriuretic peptide (NT-proBNP) | 123.0 pg/mL | 0–900 pg/mL |
| Creatine kinase (CK) | 178.0 IU/L | 25.0–195.0 IU/L |
| Creatine kinase-MB (CK-MB) | 16.6 IU/L | 2.0–25.0 IU/L |
| Lactate dehydrogenase (LDH) | 164.0 IU/L | 114.0–240.0 IU/L |
| Myoglobin (MYO) | Negative | Negative |
| **Thyroid function tests** |  |  |
| Free triiodothyronine (FT3) | 4.87 pmol/L | 3.10–6.80 pmol/L |
| Free thyroxine (FT4) | 21.50 pmol/L | 12.00–22.00 pmol/L |
| Thyroid-stimulating hormone (TSH) | 1.74 μIU/mL | 0.27–4.20 μIU/mL |
| **Vitamins** |  |  |
| Vitamin B12 | **1192.0 ng/L ↑** | 191–663 ng/L |
| Folate | **23.4 μg/L ↑** | 4.20–19.8 μg/L |
| **Amyloidosis workup** |  |  |
| Serum immunofixation electrophoresis | Negative | Negative |
| Urine immunofixation electrophoresis | Negative | Negative |
| Serum free light chain (κ) | 3.38 g/L | 1.38–3.75 g/L |
| Serum free light chain (λ) | 1.21 g/L | 0.93–2.42 g/L |
| κ/λ ratio | 2.79 | 1.17–2.93 |
| Urine microalbumin | 9.0 mg/L | 0–20.0 mg/L |
| **Coagulation function** |  |  |
| Prothrombin time (PT) | 12.90 s | 11.0–14.5 s |
| International normalized ratio (INR) | 0.96 | 0.8–1.25 |
| Activated partial thromboplastin time (APTT) | 38.6 s | 28.0–43.0 s |
| Fibrinogen (FIB) | 2.43 g/L | 2.00–4.00 g/L |
| D-dimer | 0.20 μg/mL | 0.00–0.70 μg/mL |
| **Other tests** |  |  |
| Hemoglobin A1c (HbA1c) | 5.2% | 4.2–5.9% |
| Pre-transfusion screening panel: HBV, HCV, HIV, syphilis | Negative | Negative |
| C-reactive protein (CRP) | Negative | Negative |
| Antinuclear antibody (ANA) | Negative | Negative |
| Infectious disease screening | Negative | Negative |
| Urinalysis | Negative | Negative |
| Stool routine | Negative | Negative |
